# Supplementary material for: Emerging Role of miR-21-5p in Neuron–Glia Dysregulation and Exosome Transfer Using Multiple Models of Alzheimer’s Disease
Source: Cells. 2022 Oct 26;11(21):3377. doi: 10.3390/cells11213377 (PMC9655962; doi:10.3390/cells11213377)
Supplement: Supplementary file 1 [file cells-11-03377-s001.zip › cells-1871729-supplementary.pdf]

SUPPLEMENTARY DATA

Emerging Role of miR-21-5p in Neuron–Glia Dysregulation and Exosome Transfer Using Multiple Models of Alzheimer's Disease

Cells

Gonçalo Garcia [1,2], Sara Pinto [1,3], Sofia Ferreira [1], Daniela Lopes [1], Maria João Serrador [1], Adelaide Fernandes [2,4], Ana Rita Vaz [1,2], Alexandre de Mendonça [5], Frank Edenhofer [6], Tarja Malm [7], Jari Koistinaho [7,8] and Dora Brites [1,2,*]

1 Neuroinflammation, Signaling and Neuroregeneration Lab, Research Institute for Medicines (iMed.ULisboa), Faculty of Pharmacy, Universidade de Lisboa, 1649-003 Lisbon, Portugal

2 Department of Pharmaceutical Sciences and Medicines, Faculty of Pharmacy, Universidade de Lisboa, 1649-003 Lisbon, Portugal

3 Instituto de Medicina Molecular, Faculdade de Medicina, Universidade de Lisboa, 1649-028 Lisbon, Portugal

4 Central Nervous System, Blood and Peripheral Inflammation Lab, Research Institute for Medicines (iMed.ULisboa), Faculty of Pharmacy, Universidade de Lisboa, 1649-003 Lisbon, Portugal

5 Faculty of Medicine, Universidade de Lisboa, 1649-028 Lisbon, Portugal

6 Department of Genomics, Stem Cell Biology and Regenerative Medicine, Center for Molecular Biosciences, University of Innsbruck, 6020 Innsbruck, Austria

7 A.I. Virtanen Institute for Molecular Sciences, University of Eastern Finland, 70211 Kuopio, Finland

8 Neuroscience Center, Helsinki Institute of Life Science (HiLIFE), University of Helsinki, 00014 Helsinki, Finland

* Correspondence: dbrites@ff.ulisboa.pt

Supplementary Table S1. List of primers used for mRNA/miRNA analysis by RT-qPCR.

**miRNAs**	**Target sequence (5' to 3')**
hsa-miR-124-3p	UAAGGCACGCGGUGAAUGCC
hsa-miR-125b-5p	UCCCUGAGACCCUAACUUGUGA
hsa-miR-21-5p	UAGCUUAUCAGACUGAUGUUGA
hsa-miR-146a-5p	UGAGAACUGAAUUCCAUGGGUU
hsa-miR-155-5p	UUAAUGCUAAUCGUGAUAGGGGU
hsa-miR-23a-3p	AUCACAUUGCCAGGGAUUUCC
hsa-miR-29a-5p	ACUGAUUUCUUUUGGUGUUCAG
hsa-miR-122-5p	UGGAGUGUGACAAUGGUGUUUG
hsa-miR-219a-5p	UGAUUGUCCAAACGCAAUUCU
hsa-miR-338-5p	AACAAUAUCCUGGUGCUGAGUG
UniSp6 (spike-in)	(Reference sequence)
U6	(Reference sequence)

**mRNAs** *(human except indicated)*	**Primer sequence (5' to 3')**
HMGB1	**FWD:** CATCTCAGGGCCAAACCGATA<br>**REV:** AGCAGACATGGTCTTCCACC
MHC-II	**FWD:** AGGGATTGCGCAAAAGCA<br>**REV:** TCACCTCCATGTGCCTTACAGA
CX3CR1	**FWD:** GTGGTGTGCTGACAAAGCTTGGAA<br>**REV:** TCATGGGTGCCATCGTAAGAA
CD68	**FWD:** TCAGCTTTGGATTCATGCAG<br>**REV:** AGGTGGACAGCTGGTGAAAG
TNF-α	**FWD:** AACCTCCTCTCTGCCATC<br>**REV:** ATGTTCGTCCTCCTCACA
S100B	**FWD:** TGTAGACCCTAACCCGGAGG<br>**REV:** TGCATGGATGAGGAACGCAT
iNOS	**FWD:** TCCGAGGCAAACAGCACATTCA<br>**REV:** GGGTTGGGGGTGTGGTGATGT
TREM2	**FWD:** ATGATGCGGGTCTCTACCAGTG<br>**REV:** GCATCCTCGAAGCTCTCAGACT
IL-6	**FWD:** ATGAACTCCTTCTCCACAAGC<br>**REV:** GTTTTCTGCCAGTGCCTCTTTG
IL-8	**FWD:** CTGCGCCAACACAGAAATTATTGTA<br>**REV:** TTCACTGGCATCTTCACTGATTCTT
IL-10	**FWD:** CCTGGAGGAGGTGATGCCCCA<br>**REV:** CCTGCTCCACGGCCTTGCTC
Arg-1	**FWD:** TGGAAACTTGCATGGACA<br>**REV:** AAGTCCGAAACAAGCCAA
TGF-β	**FWD:** TGCGCTTGAGATCTTCAAA<br>**REV:** GGGCTAGTCGCACAGAACT
C/EBPα	**FWD:** CAAAGCCAAGAAGTCGGTGGACAA<br>**REV:** TCATTGTGACTGGTCAACTCCAGC

PPARα	**FWD:** CTCGAGGACACCGGAGAGG<br>**REV:** CACGGAGCTGATCCCAAAGT
PPARα (mouse)	**FWD:** AACTGACGTTTGTGGCTGGTC<br>**REV:** CACCATGTTGGATGGATGTGG
PTEN (mouse)	**FWD:** AATTCCCAGTCAGAGGCGCTATGT<br>**REV:** GATTGCAAGTTCCGCCACTGAACA
SYP	**FWD:** AGTGCGCTAGAGCATTCTGG<br>**REV:** CCACCATTCTGCCTCGCTTA
GFAP	**FWD:** GAGGTTGAGAGGGACAATCT<br>**REV:** GCTTCATCTGCTTCCTGTCT
Cx43	**FWD:** GTTCAATCACTTGGCGTGAC<br>**REV:** AGTTGAGTAGGCTTGAAC
Ki-67	**FWD:** TCCTTTGGTGGGCACCTAAGACCTG<br>**REV:** TGATGGTTGAGGCTGTTCCTTGATG
GAPDH	**FWD:** CGCTCTCTGCTCCTCCTGTT<br>**REV:** CCATGGTGTCTGAGCGATGT
β-actin	**FWD:** CAGAGCCTCGCCTTTGCCGA<br>**REV:** ATCCATGGTGAGCTGGCGGC

hsa, Homo sapiens; miRNA(miR), microRNA; mRNA, messenger RNA; RT-qPCR, real-time quantitative polymerase chain reaction; FWD, forward; REV, reverse; HMGB1, high mobility group box protein 1 coding gene; MHC-II, major histocompatibility complex class II coding gene; CX3CR1, CX3C chemokine receptor 1 gene; CD68, cluster of differentiation 68 gene; S100B, S100 calcium-binding protein B coding gene; TNFα, tumor necrosis factor alpha coding gene; iNOS, nitric oxide synthase coding gene; TREM2, triggering receptor expressed on myeloid cells 2 coding gene; IL-6, interleukin 6 coding gene; IL-8, interleukin 8 coding gene; IL-10, interleukin 10 coding gene; Arg-1, arginase-1 coding gene; TGF-β, transforming growth factor beta coding gene; C/EBPα, CCAAT enhancer binding protein alpha coding gene; PPARα, peroxisome proliferator-activated receptor alpha gene; PTEN, phosphatase and tensin homolog; SYP, synaptophysin gene; GFAP, glial fibrillary acidic protein; Cx43 (GJA1), connexin 43 (gap junction protein alpha 1); Ki-67 (MKI67), marker of proliferation Ki-67 gene; GAPDH, glyceraldehyde-3-phosphate dehydrogenase coding gene; β-actin coding gene.

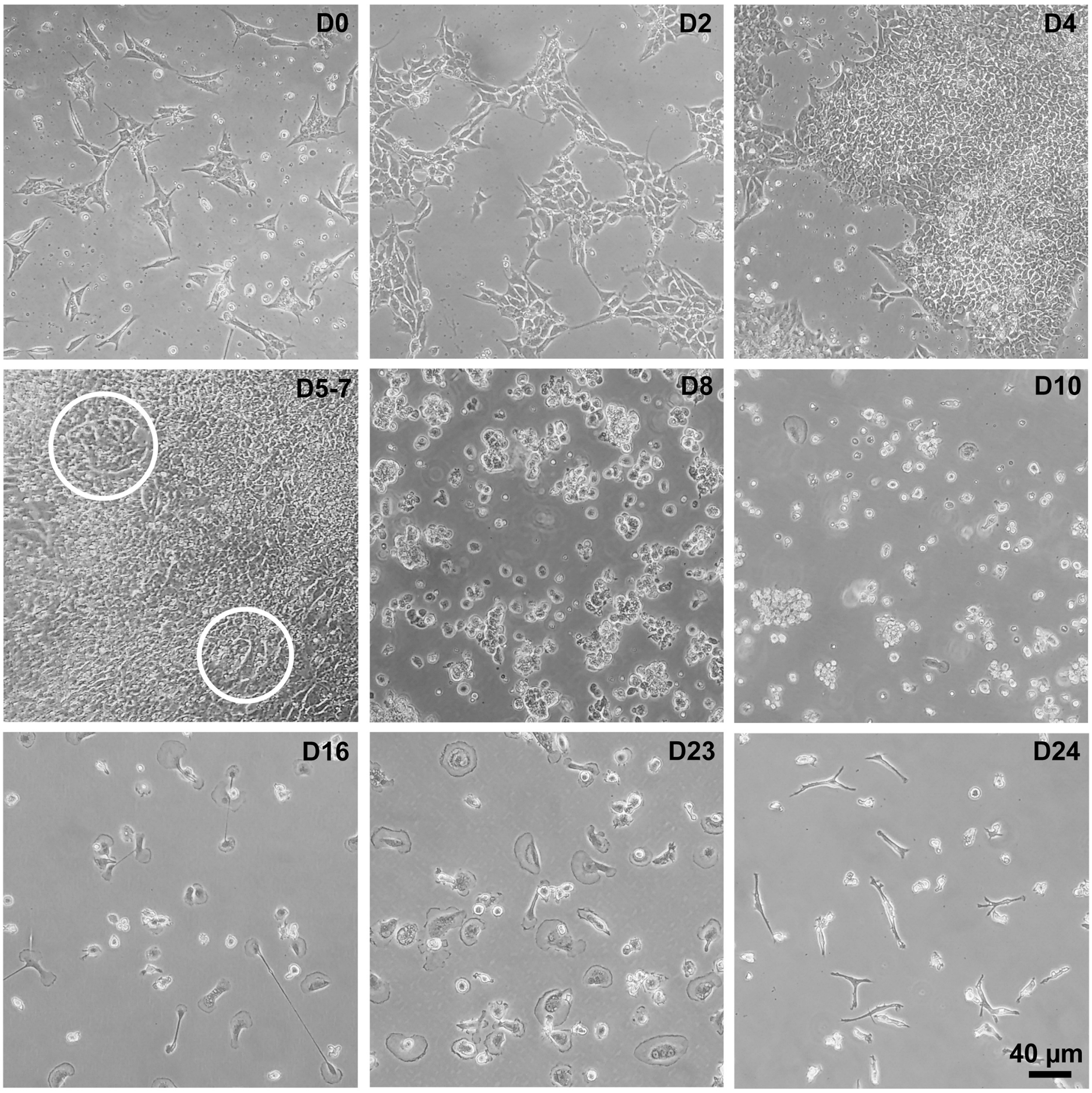

Supplementary Figure S1. Representative brightfield images of the sequential differentiation stages associated to the differentiation protocol of microglia, in Figure 4 of the manuscript, and as described in Material and Methods. First, we induced early mesodermal differentiation from day 0 (D0) to D2, in low oxygen conditions. Then, at D2, we induced hematovascular mesodermal differentiation until D4, when we cultured cells back on normoxia to start the hemogenic endothelial differentiation. From D5 to D7, Hematopoietic progenitor cells (HPCs, highlighted in white circles) were expanded. At D8, myeloid progenitors were suspended, cultured in ultra-low attachment (ULA) plates, and expanded for 2 days. At D10, we started the primitive microglia differentiation until D16 to D23. Then at D24, primitive microglia were detached and plated into poly-D-lysine-coated plates and submitted to experiments. All images are at the same magnification and scale bar corresponds to 40 µm. D, days in culture.

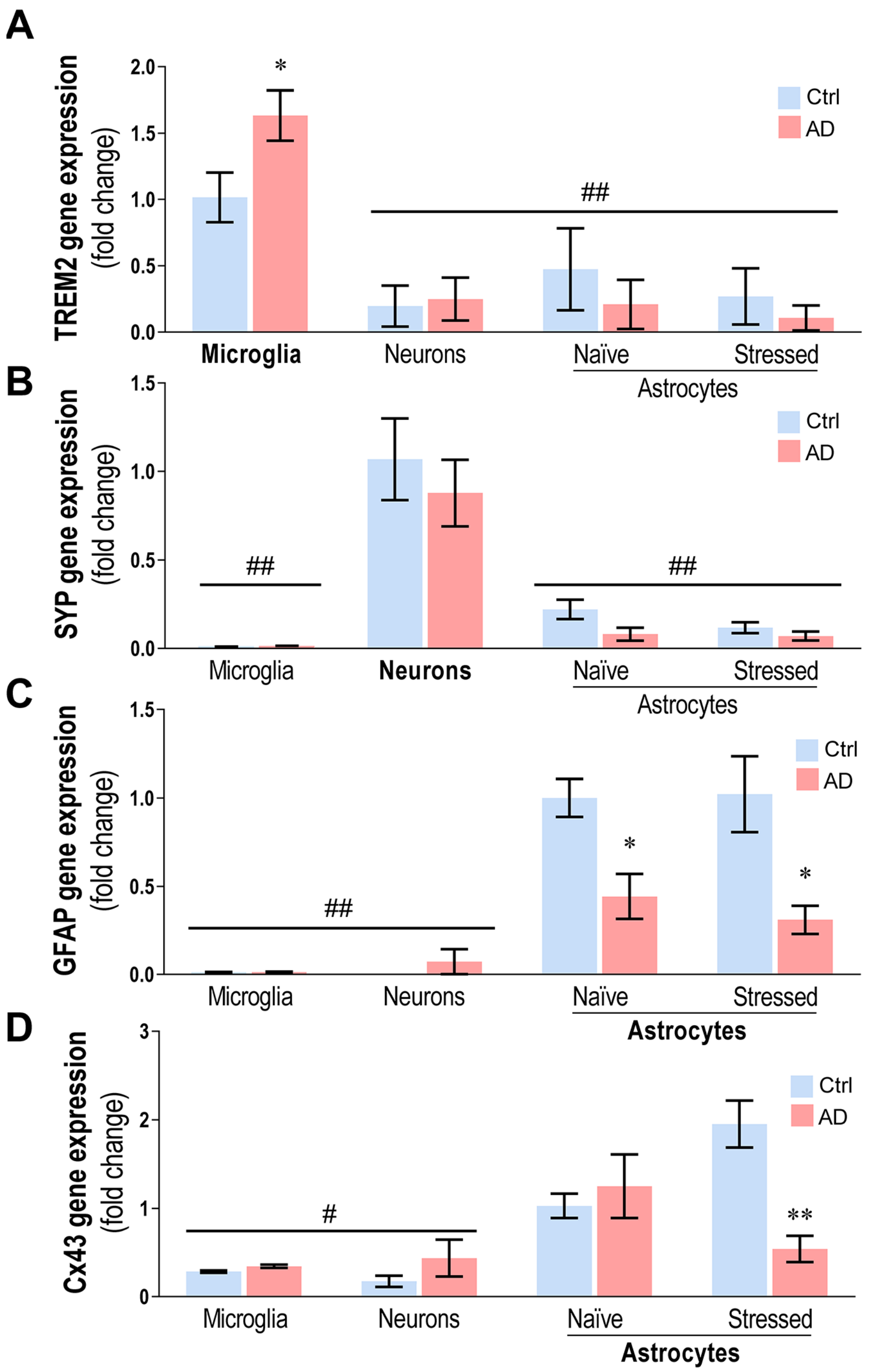

Supplementary Figure S2. Characteristic markers of microglia, neurons and astrocytes differentiated from induced pluripotent stem cells (iPSCs) support that each cell differentiation was successively achieved. (A) Gene expression of TREM2 (microglial marker) in iPSC-derived cells *vs.* Ctrl microglia; (B) Gene expression of SYP (neuronal marker) in iPSC-derived cells *vs.* Ctrl neurons; (C) Gene expression of GFAP, and (D) gene expression of Cx43 (astrocyte markers) in iPSC-derived cells *vs.* Ctrl naïve astrocytes. Results are mean ± SEM from four independent experiments, presented in fold change. Two-tailed student's *t*-test: * $p < 0.05$; ** $p < 0.01$ *vs.* respective Ctrl paired cell type; One-way ANOVA # $p < 0.05$; ## $p < 0.05$ *vs.* each marker-specific cell type. TREM2, triggering receptor expressed on myeloid cells 2 coding gene; SYP, synaptophysin coding gene; GFAP, glial fibrillary acidic protein coding gene; Cx43 (or GJA1), connexin 43 (or gap junction protein alpha 1) coding gene; Ctrl, control; Naïve, non-immunostimulated astrocytes; stressed, immunostimulated with a cocktail composed by complement component C1q + interleuklin-1 alpha + tumor necrosis factor alpha.

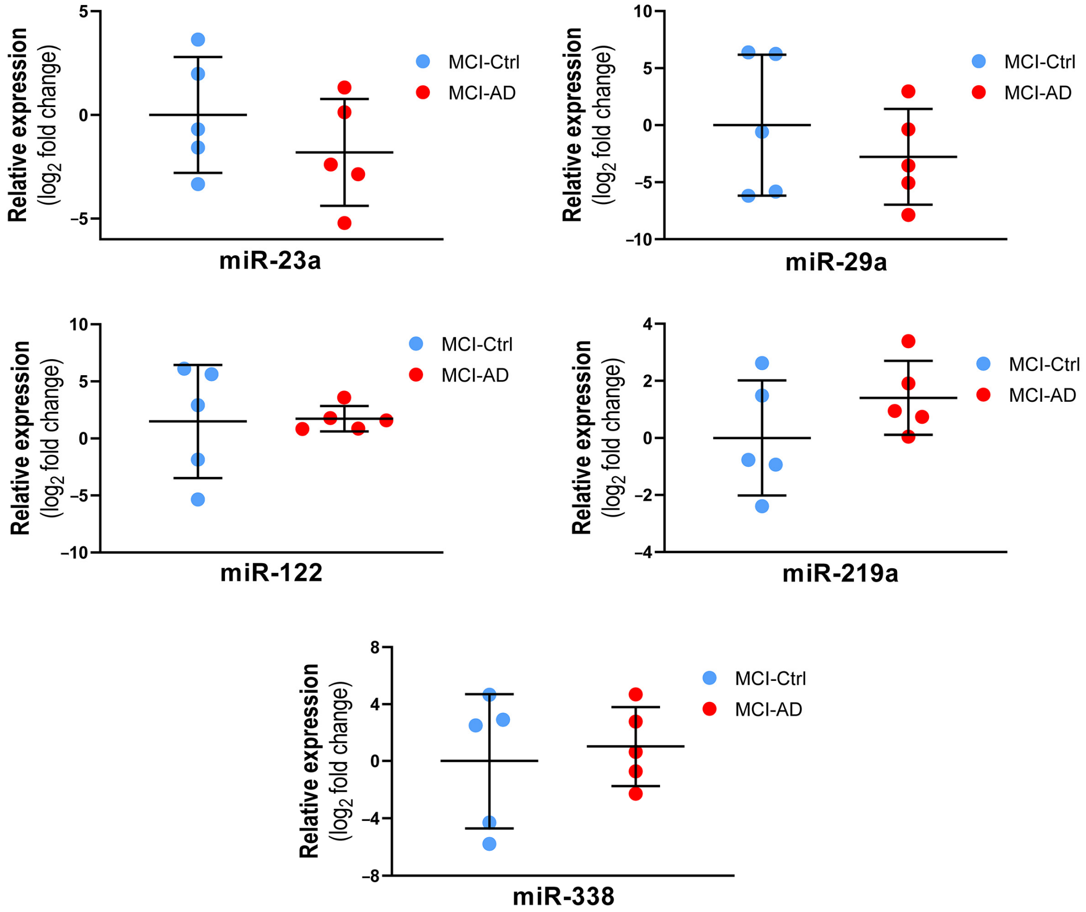

Supplementary Figure S3. Additional set of AD-associated miRNAs quantified in the CSF from MCI patients who fulfilled criteria for MCI due to AD (MCI-AD) *vs*. MCI patients with no biomarker criteria for AD (MCI-Ctrl). miR-23a, miR-29a, miR-122, miR-219a and miR-338 profile in the CSF samples from MCI-AD (red dots) side-by-side with miRNA profile in the CSF from MCI-Ctrl (blue dots). Results are mean ± SEM from five different subjects of each group, presented in binary logarithm of fold change. Mann-Whitney U test with Bonferroni post-hoc correction: ** $p < 0.01$ MCI-AD *vs*. MCI-Ctrl. AD, Alzheimer's disease; Ctrl, control; MCI, mild cognitive impairment; CSF, cerebrospinal fluid.
